# Removal of Hexavalent Chromium(VI) from Wastewater Using Chitosan-Coated Iron Oxide Nanocomposite Membranes

**DOI:** 10.3390/toxics10020098

**Published:** 2022-02-19

**Authors:** Jung Eun Park, Jun-Ho Shin, Wonzin Oh, Sang-June Choi, Jeongju Kim, Chorong Kim, Jongho Jeon

**Affiliations:** 1 Department of Applied Chemistry, College of Engineering, Kyungpook National University, Daegu 41566, Korea; pje1204@knu.ac.kr (J.E.P.); sa011107@knu.ac.kr (J.-H.S.); 2 School of Architectural, Civil, Environmental, and Energy Engineering, Kyungpook National University, Daegu 41566, Korea; wonzin@knu.ac.kr (W.O.); sjchoi@knu.ac.kr (S.-J.C.); 3 Korea Hydro & Nuclear Power Co., Ltd., Central Research Institute, Daejeon 34101, Korea; jeongju.kim@khnp.co.kr (J.K.); chorong.kim@khnp.co.kr (C.K.)

**Keywords:** composite membrane, filtration, immobilization, water treatment, hexavalent chromium

## Abstract

Chromium is a toxic and carcinogenic heavy metal that originates from various human activities. Therefore, the effective removal of chromium from aqueous solutions is an extremely important global challenge. Herein, we report a chitosan-coated iron oxide nanoparticle immobilized hydrophilic poly(vinylidene) fluoride membrane (Chi@Fe_2_O_3_–PVDF) which can potentially be used for efficient removal of hexavalent chromium(VI) by a simple filtration process. Membrane filtration is an easy and efficient method for treating large volumes of water in a short duration. The adsorption experiments were conducted by batch and continuous in-flow systems. The experimental data showed rapid capture of hexavalent chromium (Cr(VI)) which can be explained by the pseudo-second-order kinetic and Langmuir isotherm model. The nanocomposite membrane exhibited high adsorption capacity for Cr(VI) (14.451 mg/g in batch system, 14.104 mg/g in continuous in-flow system). Moreover, its removal efficiency was not changed significantly in the presence of several competing ions, i.e., Cl^−^, NO_3_^−^, SO_4_^2−^, and PO_4_^3−^. Consequently, the Chi@Fe_2_O_3_-PVDF-based filtration process is expected to show a promising direction and be developed as a practical method for wastewater treatment.

## 1. Introduction

Environmental pollution caused by leakage of heavy metals is a serious problem worldwide [1,2]. Chromium is a toxic heavy metal that commonly exists in wastewater produced during steel manufacturing, leather tanning, and so on. Particularly, hexavalent chromium (Cr(VI)) (e.g., HCrO_4_^−^) is generated in the process of various industrial processes and is 500 times more toxic than trivalent Cr(III), which is also present in nature [3]. This metal species can also be generated in the chemical decontamination procedure for removal of the oxide layer deposited in the primary system of a nuclear power plant. Moreover, radioactive chromium (^51^Cr, t_1/2_ = 27.7 days) is found in radioactive wastewater as a result of several nuclear activities, such as radioisotope production and radiochemistry research [4,5]. Cr(VI) is known to cause many human diseases, such as anemia, liver damage, diarrhea, cancer, and kidney damage. The World Health Organization has established 0.05 mg/L as the maximum admissible concentration of Cr(VI), and 2 mg/L of total chromium in drinking water [6,7,8]. Therefore, an efficient method for desalination of hexavalent chromium from wastewater is necessary.

For years, many methods have been reported for the removal of Cr(VI), such as electrochemical treatment [9,10,11], reverse osmosis [12,13], photocatalytic reduction [14], ion exchange [15,16], and adsorption [17,18,19,20,21]. Among them, the adsorption method has the advantage of a simple and fast removal process and thus, it has high potential for removing chromium ions from wastewater. Various types of adsorbents have been developed recently. For example, functionalized iron oxide nanoparticles (i.e., Fe_2_O_3_ and Fe_3_O_4_) have been widely used for efficient removal of toxic heavy metal ions due to their low toxicity, easy and inexpensive preparation, and large surface area [22,23,24,25]. However, their adsorption efficiency is not satisfactory due to the slow adsorption kinetics, and moreover an additional separation process for removal of the adsorbent from the water is required after the desalination procedure is finished. In this study, we developed an adsorbent-embedded polymeric membrane for rapid capture of Cr(VI) by a filtration process. Compared to previous methods, membrane filtration can be a highly effective technique for removing heavy metal ions from large volumes of water as it does not require an additional process of removing adsorbents and can quickly and effectively remove pollutants from wastewater [26]. The use of a composite membrane, which stably immobilizes adsorbents, minimizes the aggregation of the nanoadsorbent and allows for easy elimination of contaminated solid waste. In addition, membrane filtration is suitable for continuous systems [27,28] and adsorbent-immobilized membranes can be applied in a continuous in-flow system for the efficient treatment of wastewater [29]. Among various polymeric membranes, we selected hydrophilic poly(vinylidene fluoride) (PVDF), a commercially available polymeric membrane, for its advantages, such as thermal stability, chemical resistance, high mechanical strength, and tunable hydrophilicity. Due to these characteristics, it is widely used in various membrane filtration processes [30,31]. Chitosan is widely used for water purification because of its properties, such as biodegradability and nontoxicity. It has large numbers of amine functional groups that can chelate the metal ions easily [32].

Previously, we have reported continuous in-flow removal of radioactive wastes in water using an adsorbent-immobilized composite membrane [33,34]. The gold nanoparticle immobilized cellulose acetate membrane showed rapid adsorption capability for radioactive iodide ions within a short duration. In addition, the nanoparticles immobilized to the membrane were highly stable, due to which leaching did not occur even under severe conditions, such as 0.1 M HCl, 0.1 M NaOH, and 1.0 M NaCl. Inspired by the previous research, a new composite adsorbent can be fabricated by immobilizing chitosan-coated iron oxide on the hydrophilic poly(vinylidene fluoride) (Chi@Fe_2_O_3_–PVDF) for rapid, selective, and efficient removal of Cr(VI). In the present study, the applicability of the composite membrane to Cr(VI) removal from various real water samples and the performance of the composite membrane in the presence of coexisting ions were investigated.

## 2. Materials and Methods

### 2.1. General Methods

Chitosan-coated iron oxide nanomaterials (γ-Fe_2_O_3_, average hydrodynamic diameter: 50 nm) were purchased from Chemicell (Berlin, Germany). Potassium dichromate (K_2_Cr_2_O_7_, 99%) was purchased from Sigma Aldrich (Yongin, Korea). Hydrochloric acid (HCl, 37%), sodium hydroxide (NaOH), acetic acid (CH_3_CO_2_H, 99%), acetone, sulfuric acid (H_2_SO_4_, 97%), and 1,5-diphenylcarbazide (DPC, C_13_H_14_N_4_O, 99%) were purchased from Duksan Pure Chemicals Co. Ltd. (Daejeon, Korea). All reagents were of analytical grade and used without further purification. Hydrophilic poly(vinylidene fluoride) membrane (PVDF, pore size = 0.20 μm, diameter = 47 mm) was purchased from Hyundai Micro Co. Ltd. (Daejeon, Korea). The vacuum filtration apparatus was purchased from Phenomenex Inc. (Torrance, CA, USA).

### 2.2. Preparation of the Composite Membrane (Chi@Fe_2_O_3_–PVDF)

Chi@Fe_2_O_3_–PVDF was prepared as described in Figure S1 with a glass vacuum filter assembly. First, 2.2 mg Chi@Fe_2_O_3_ was dissolved in 5 wt% acetic acid solution with a final volume of 100 mL. The hydrophilic PVDF membrane (diameter = 47 mm, pore size = 0.20 μm) was placed between the filter holder fritted glass support and the graduated funnel. Chi@Fe_2_O_3_ nanoparticles (100 mL) were poured into the graduated funnel and then a vacuum was applied until all the nanoparticles passed through the membrane. Next, a protonation process was conducted to effectively use the amino groups of the Chi@Fe_2_O_3_–PVDF for Cr(VI) adsorption. The composite membranes were treated with 0.1 M HCl for 30 min at 25 °C. After washing the membrane with DI water several times, the prepared Chi@Fe_2_O_3_–PVDF was kept under ambient conditions until it was applied in the desalinization experiment.

### 2.3. Analytical Instruments and Characterization of the Composite Membrane (Chi@Fe_2_O_3_–PVDF)

Concentrations of Cr(VI) in aqueous solutions were measured using quartz cuvettes and a UV-Vis spectrophotometer (UV-1800, Shimadzu, Japan) (Figures S2 and S3). The surface charge of the PVDF membrane and Chi@Fe_2_O_3_ were analyzed using the streaming potential method in an Electro-Kinetic analyzer (Anton Paar GmbH, Surpass 3, Seoul, Korea). The elemental composition of the Chi@Fe_2_O_3_–PVDF was analyzed by scanning electron microscopy with energy-dispersive X-ray (SEM-EDX, SU8220, Hitachi, Japan) analysis with accelerating voltages of up to 30 kV. EDX spectra were recorded in area scanning mode by focusing the electron beam onto a region of the sample surface. The amount of iron particles liberated from the composite membrane was measured by an inductively coupled plasma spectrometer (ICP, Optima 7300DV, Perkin Elmer, UK).

### 2.4. Adsorbate Preparation

A stock solution of Cr(VI) (500 mg/L) was prepared by the dissolution of the analytical reagent K_2_Cr_2_O_7_ in 50 mL of DI water, and further dilution was conducted to obtain the desired concentrations. The initial pH adjustment was made using 0.1 M HCl or 0.1 M NaOH solutions as required. The pH was measured using a pH meter. All the adsorption experiments were performed at solution pH 4, except for the effect of pH, which was performed by varying the pH from 2 to 10.

### 2.5. Adsorption Experiments

#### 2.5.1. Adsorption Experiments Using Batch System

To investigate the performance of the Chi@Fe_2_O_3_–PVDF for removal of Cr(VI), K_2_Cr_2_O_7_ was diluted with 3 mL DI water at different concentrations (1, 5, 10, 25, 50, and 100 ppm) and poured into Petri dishes (50-mm diameter and 15-mm height). Chi@Fe_2_O_3_–PVDF was immersed in each Petri dish and kept at room temperature under gentle shaking. The solution was sampled (200 µL) at different time periods (1, 3, 5, 10, and 30 min). The amount of adsorbed Cr(VI) on the membrane was analyzed by the standard diphenylcarbazide method (540 nm) (0.03 to 1 ppm) [35] and the direct method (370 nm) (above 1 ppm) [36] using UV-Vis spectrometry (Figures S2 and S3).

The percentage removal efficiency of Chi@Fe_2_O_3_–PVDF was determined using Equation (1):

(1) $$\mathrm{Removal}\;\mathrm{efficiency}\;\left(\%\right)=\frac{\left(C_{0}-C_{e}\right)}{C_{0}}\times 100$$

where Q_e_ (mg/g), equilibrium adsorption capacity of Chi@Fe_2_O_3_–PVDF, was determined using Equation (2):

(2) $$Q_{e}=\frac{\left(C_{0}-{\;C}_{e}\right)}{m}\times V$$

where Q_e_ (mg/g) is the quantity of Cr(VI) that was adsorbed on the Chi@Fe_2_O_3_–PVDF at equilibrium time. C_0_ (mg/L) and C_e_ (mg/L) represent the initial and final concentration of Cr(VI) in the aqueous solution at time t, V (L) is the volume of the Cr(VI) solution, and m (g) represents the mass of the adsorbents (Chi@Fe_2_O_3__,_ 2.2 mg).

#### 2.5.2. Adsorption Experiments Using Continuous-Flow System

A stock solution of K_2_Cr_2_O_7_ (500 mg/L) was diluted with pure water to obtain various concentrations (0.5, 1, 2.5, 5, and 10 ppm) of Cr(VI) solution. Chi@Fe_2_O_3_–PVDF was placed between the filter holder fritted glass support and the graduated funnel. Next, the Cr(VI) solution was poured into the graduated funnel and a vacuum was applied until the entire solution was passed through the Chi@Fe_2_O_3_–PVDF. The concentration of adsorbed Cr(VI) on the membrane was analyzed using UV-Vis spectrometry.

#### 2.5.3. Adsorption Isotherms

The adsorption isotherm was determined using the Cr(VI) solution at 25 °C. Briefly, Chi@Fe_2_O_3_–PVDF was treated with 3 mL (batch system) or 50 mL (continuous-flow system) Cr(VI) at different initial concentrations (1–100 ppm in the batch system, 0.5–10 ppm in the continuous-flow system). The final concentration of Cr(VI) after the adsorption process was measured via UV-Vis spectroscopy. The Langmuir and Freundlich isotherm models were applied to describe the equilibrium adsorption using Equations (3) and (4), respectively:

(3) $$\mathrm{Langmuir}\;\mathrm{equation}:\;\frac{C_{e}}{Q_{e}}=\frac{C_{e}}{Q_{\max}}+\frac{1}{Q_{\max}{\;K}_{L}}$$

(4) $$\mathrm{Freundlich}\;\mathrm{equation}:\;\ln Q_{e}=\ln K_{F}+\frac{1}{n}\ln C_{e}$$

where Q_max_ (mg/g) is the maximum adsorption capacity of the adsorbents (Chi@Fe_2_O_3_). K_L_ and K_F_ are the Langmuir and Freundlich adsorption constants, respectively.

#### 2.5.4. Adsorption Kinetics

The adsorption kinetics of Cr(VI) were determined using 25 ppm of Cr(VI) solution at room temperature. Briefly, 3 mL Cr(VI) (25 μM) solution was shaken with Chi@Fe_2_O_3_–PVDF. At different time periods (1, 3, 5, 10, 30, and 60 min), the Cr(VI) solution (200 μL) was collected and the concentration of Cr(VI) was determined via UV–Vis spectroscopy by measuring the absorbance variation at the maximum wavelength. The adsorption capacity was fitted into the pseudo-first-order and pseudo-second-order kinetics equations with respect to time, as expressed in Equations (5) and (6), respectively:

(5) $$\mathrm{Pseudo}\text{-}\mathrm{first}\text{-}\mathrm{order}\;\mathrm{kinetic}\;\mathrm{model}:\;\ln\left(Q_{e}-Q_{t}\right)=\ln Q_{e}-\frac{k_{1}t}{2.303}$$

(6) $$\mathrm{Pseudo}\text{-}\mathrm{second}\text{-}\mathrm{order}\;\mathrm{kinetic}\;\mathrm{model}:\;\frac{t}{Q_{t}}=\frac{1}{k_{2}Q_{e}^{2}}+\frac{t}{Q_{e}}$$

where Q_e_ and Q_t_ are the quantities of Cr(VI) (mg/g) at equilibrium and time t, respectively. k_1_ (min^−1^) and k_2_ (g mg^−1^ min^−1^) are the pseudo-first-order and pseudo-second-order adsorption rate constants, respectively.

## 3. Results and Discussion

### 3.1. Characterization of Nanocomposite Membrane

**Figure 1:**
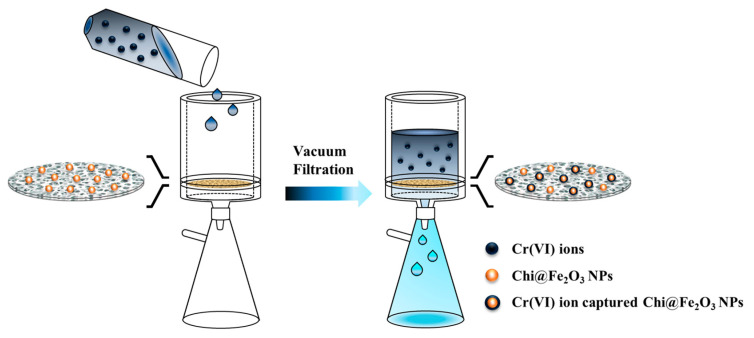
Schematic illustration of the desalinization procedure using a chitosan-coated iron oxide nanocomposite poly(vinylidene fluoride) membrane (Chi@Fe_2_O_3_–PVDF).

**Figure 2:**
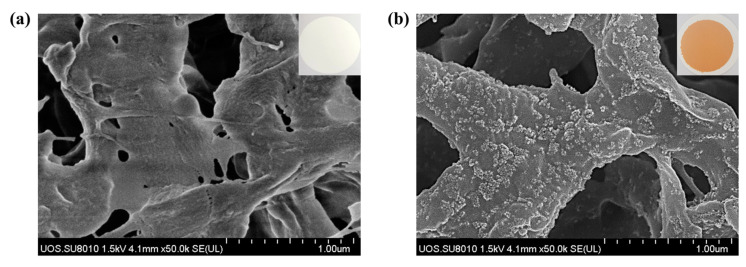
Scanning electron microscope images of (**a**) poly(vinylidene fluoride) (PVDF); (**b**) Chi@Fe_2_O_3_–PVDF. White dots indicate Chi@Fe_2_O_3_ NPs deposited on the membrane.

**Figure 3:**
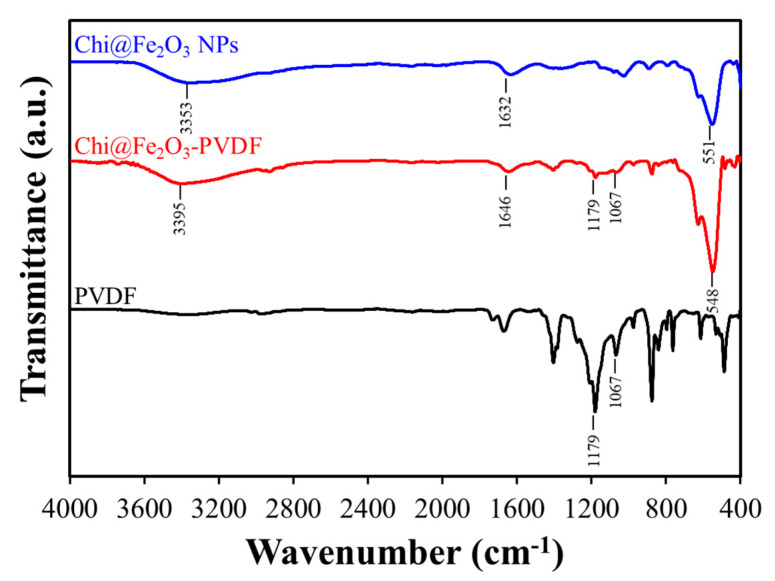
Fourier-transform infrared spectra (FT-IR) of poly(vinylidene fluoride) (PVDF) (black), Chi@Fe_2_O_3_ NPs (blue), and Chi@Fe_2_O_3_–PVDF (red).

The main strategy for desalination of Cr(IV) by using a nanocomposite membrane and filtration system was illustrated in Figure 1. The preparation of the nanoadsorbent-embedded polymeric membrane(Chi@Fe_2_O_3_–PVDF) is shown in Figure S1. The immobilization of chitosan-coated iron oxide nanoparticles was conducted using commercially available hydrophilic PVDF membrane. Using a vacuum filtration system, 2.2 mg chitosan-coated iron oxide nanoparticles were stably immobilized on the membrane, which exhibited a homogeneous and yellow-brown color. The surface morphology was characterized using scanning electron microscopy (SEM) and energy-dispersive X-ray analysis (EDX). In Figure 2, the chitosan-coated iron oxide nanoparticles were well distributed on the nanofibers of the PVDF membrane. To confirm whether the nanoparticles were stably immobilized on the membrane and could be used for chromium removal, ICP analysis was performed under various conditions (e.g., deionized water; Cr(VI) 25 ppm (pH 4, 6, and 10); 0.1 M HCl; 0.1 M NaOH; and 0.1 M NaCl) after immersing the membrane for 1 h. As shown in Table 1, 0.371% of iron oxide nanoparticles were released from the membrane under 0.1 M HCl conditions. In addition, it was observed that 0.033% and 0.046% nanoparticles were lost in the 25 ppm chromium solutions under pH 7 and pH 10, respectively. Not only are these very small amounts, but also their loss is not expected to have a significant impact because the filtration process for removing chromium ions proceeds within about 20 s. These results can be explained in two ways. First, the stability of composite nanomaterials can be explained by the electrostatic interaction between the chitosan-coated iron oxide nanoparticles (zeta potential = +21.2 mV) and the PVDF membrane (zeta potential = −18.9 mV). At neutral pH, the positively charged amine functional group of chitosan and negatively charged membrane have strong electrostatic interactions. Second, dipole interactions between electron-poor methylene (CH_2_) groups in the PVDF chain and hydroxy groups on the surface of the iron oxide may contribute to stability. Figure 3 shows the Fourier-transform infrared (FT-IR) spectra of PVDF, Chi@Fe_2_O_3_ NPs, and Chi@Fe_2_O_3_–PVDF. In the spectra of Chi@Fe_2_O_3_–PVDF, the bands at 3395, 1646, and 548 cm^−1^ are characteristics of the stretching vibrations of –OH, N–H bending of NH_2_, and Fe–O stretching vibration of Fe_2_O_3_ in Chi@Fe_2_O_3_ NPs, respectively. Additionally, the bands at 1179, and 1067 cm^−1^ indicate the stretching vibration of –CF_2_ and the stretching band of C–F in PVDF. These results show that the composite membrane is fabricated successfully using PVDF and Chi@Fe_2_O_3_ NPs.

**Figure 4:**
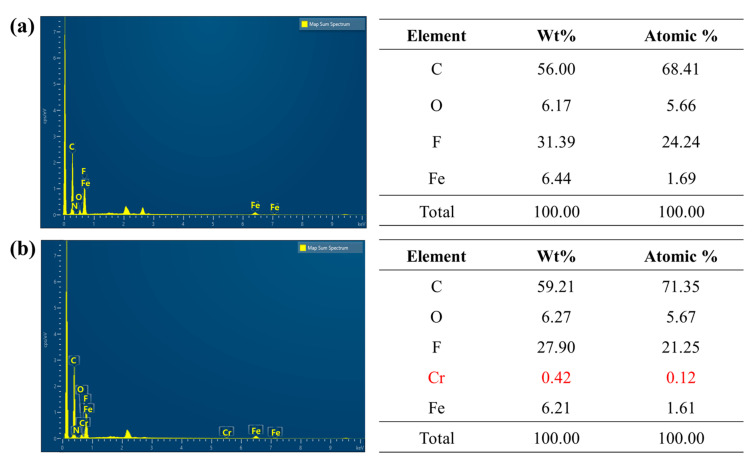
Energy-dispersive X-ray spectroscopy analysis of (**a**) Chi@Fe_2_O_3_–PVDF and (**b**) Cr(VI) captured Chi@Fe_2_O_3_–PVDF.

Elemental analysis of the Chi@Fe_2_O_3_–PVDF showed an iron atom peak, representing the incorporation of PVDF membrane and the nanoadsorbent (Figure 4a). Carbon, oxygen, and fluorine atoms were observed and attributed to the backbone structure of the PVDF membrane. These results demonstrate that Chi@Fe_2_O_3_–PVDF was successfully prepared.

### 3.2. Effect of Solution pH

**Figure 5:**
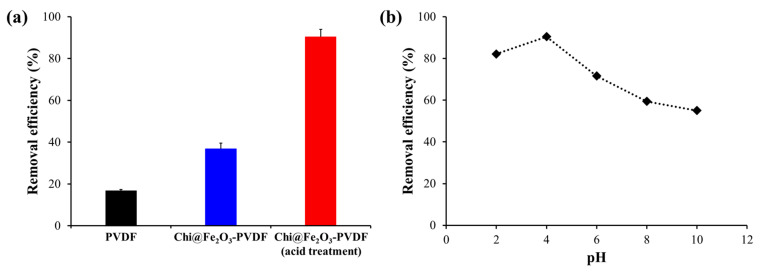
(**a**) Comparison of removal efficiency of PVDF, Chi@Fe_2_O_3_–PVDF, and Chi@Fe_2_O_3_–PVDF after acid treatment; (**b**) Effect of pH on the adsorption of Cr(VI) using Chi@Fe_2_O_3_–PVDF.

pH is an important factor in the adsorption process of Chi@Fe_2_O_3_–PVDF because the amino groups of chitosan can be protonated at lower pH, which affects the surface charge of Chi@Fe_2_O_3_–PVDF. We first investigated the removal capability of the composite membrane under some different conditions. As shown in Figure 5a, Chi@Fe_2_O_3_–PVDF pretreated with an acidic solution had the highest removal efficiency, which is related to the positively charged surface caused by the amino group of protonated chitosan. In addition, the surface charge of nanoadsorbents depends on the pH of solvent. The surface charge becomes neutral at pH 5.8 for iron oxide; further lowering the pH will render the adsorbent surface rich in positive charges and Cr(VI), resulting in better adsorption [37,38]. On the other hand, non-treated composite membrane and bare PVDF exhibited much lower removal efficiencies than that of pretreated Chi@Fe_2_O_3_–PVDF (Figure 5a). Next, the removal of Cr(VI) was evaluated by immerging the composite membrane, which was pretreated under acidic condition, in Cr(VI) solutions in varied pH. Figure 5b shows the result of testing the removal efficiency by varying the pH of the Cr(VI) solution. Cr(VI) removal efficiency gradually increased as the pH decreased from 10 to 4 and reached its peak at pH 4 (90.45%). However, at pH 2, the removal efficiency of Cr(VI) decreased. This is related to the conversion of the ionic form of Cr(VI) with varying pH value. Cr(VI) anions have different ionic forms depending on the pH, where CrO_4_^2−^ is dominant at pH 6 or higher, and HCrO_4_^−^ and Cr_2_O_7_^2−^ are the main forms at pH 2–6. H_2_CrO_4_ is predominant at more acidic pH. In addition, as the pH value of the iron oxide increases, a negative charge is generated on the surface of the adsorbents and thus, the electrostatic repulsion between the iron oxides and the Cr(VI) anion increases significantly. Therefore, Chi@Fe_2_O_3_–PVDF has the highest removal efficiency at pH 4 for Cr(VI) by electrostatic interactions among chitosan, iron oxide surfaces, and Cr(VI).

### 3.3. Adsorption of Cr(VI) Using Chi@Fe_2_O_3_–PVDF

#### 3.3.1. Batch System

**Figure 6:**
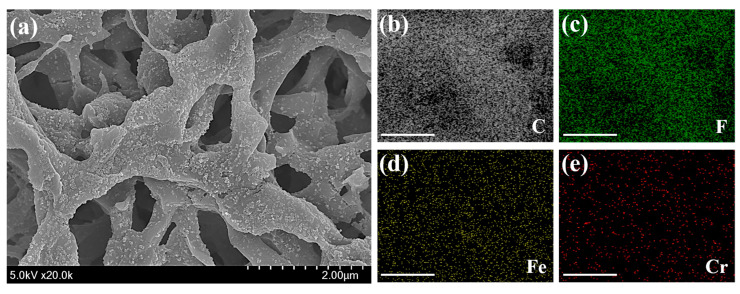
(**a**) Scanning electron microscope image of Cr(VI) captured Chi@Fe_2_O_3_–PVDF; (**b–e**) Energy-dispersive X-ray spectroscopy elemental mapping patterns of Cr(VI) captured Chi@Fe_2_O_3_–PVDF (scale bar = 1 μm).

**Figure 7:**
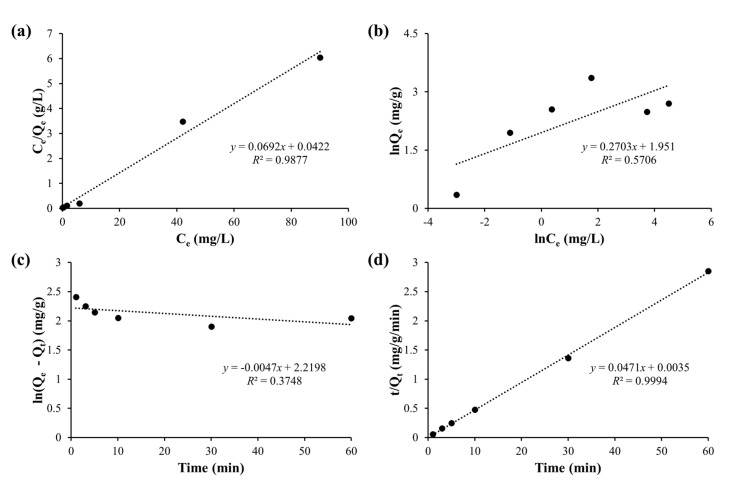
The adsorption isotherms of Cr(VI) fitted on the basis of (**a**) the Langmuir model; (**b**) the Freundlich model; (**c**) the pseudo-first-order adsorption kinetic; and (**d**) the pseudo-second-order adsorption kinetic.

To investigate the desalinization capability of the Chi@Fe_2_O_3_–PVDF, it was immersed in aqueous solutions containing Cr(VI) at pH 4 to measure the typical desalinization performance of Chi@Fe_2_O_3_–PVDF for Cr(VI) with 30 min by batch system. The removal capacity (Q_e_) of the Chi@Fe_2_O_3_–PVDF increased with an increase in the initial concentration of Cr(VI). The adsorption of Cr(VI) on the surface of Chi@Fe_2_O_3_–PVDF was confirmed by the EDX analysis that shows a characteristic Cr peak (Figure 4b). The elemental mapping patterns of Chi@Fe_2_O_3_–PVDF showed the presence of iron with chromium, thus confirming capture of the target metal species on the composite membrane (Figure 6). The linear fitting of the observed data based on the Langmuir and Freundlich isotherm models (Figure 7a,b) revealed that the performance of Chi@Fe_2_O_3_–PVDF was better fitted by the Langmuir equation with a correlation factor (R^2^) of 0.9877. These results indicated the monolayer adsorption mechanism, and the observed maximum adsorption capacity (Q_max_) obtained using Equation (3) was 14.451 mg/g. The corresponding parameters for these models are summarized in Table 2.

The kinetic parameters of batch adsorption are analyzed to evaluate the adsorbent. The removal efficiency of Cr(VI) was determined as a function of time (1–60 min) to determine the optimum time for the desalinization experiments. The adsorption of Cr(VI) was rapid in the initial 10 min, and gradually became slower, and finally reached the equilibrium after 30 min. The kinetic results of the pseudo-first-order and pseudo-second-order kinetic models are shown in Figure 7c,d. Based on the calculated kinetic parameters, the pseudo-second-order kinetic model fitted better with the kinetic results, which exhibited an R^2^ value for the pseudo-second-order kinetic model that is approximately 1 (0.9994) and higher than that of the pseudo-first-order kinetic model (0.3748). In addition, the theoretical Q_max_ for Cr(VI) obtained from the pseudo-second-order kinetic model is closer to the experimental Q_max_ value. These results indicate that the adsorption behavior is well described by the pseudo-second-order kinetics, suggesting that the rate-limiting step is surface adsorption and involves chemisorption mechanism.

#### 3.3.2. Continuous In-Flow System

**Figure 8:**
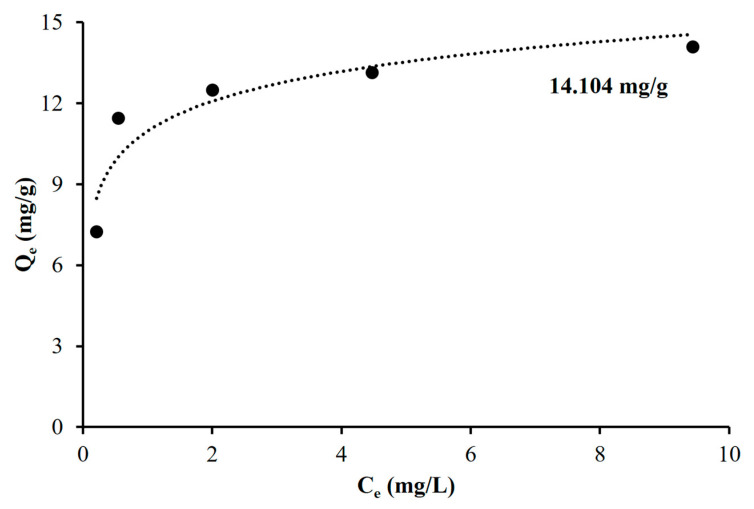
Isotherm data for the removal of Cr(VI) using Chi@Fe_2_O_3_–PVDF in the continuous in-flow system.

To study the adsorption behavior of Chi@Fe_2_O_3_–PVDF for Cr(VI) in the continuous in-flow system, the data tested in the vacuum filtration system were fitted to the Langmuir model and the Freundlich model. As shown in Figure S4, the linear fitting of the observed data based on the Langmuir and Freundlich isotherm models revealed that Chi@Fe_2_O_3_–PVDF was better estimated by the Langmuir model, with the R^2^ value of 0.995. In addition, the maximum adsorption capacity of Chi@Fe_2_O_3_–PVDF in the continuous in-flow system was confirmed to be 14.104 mg/g by the Langmuir model (Figure 8).

### 3.4. Effect of Coexisting Ions on Removal Efficiency in Real Water Samples

**Figure 9:**
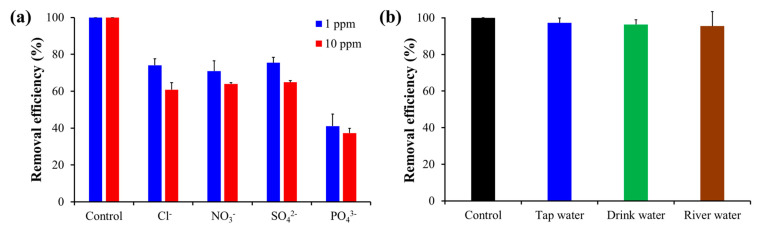
(**a**) Effect of coexisting ions on the removal of Cr(VI) using Chi@Fe_2_O_3_–PVDF with a volume of 50 mL, pH value of 4, and Cr(VI) concentration of 1 ppm; (**b**) Real water experiments for the removal of Cr(VI) using Chi@Fe_2_O_3_–PVDF with a volume of 50 mL, pH value of 4, and Cr(VI) concentration of 0.1 ppm.

Chromium in actual environmental water generally exists with various types of ions. Therefore, to measure the effect of coexisting ions on chromium removal, experiments were conducted in the presence of four types of ions, i.e., Cl^−^, NO_3_^−^, SO_4_^2−^, and PO_4_^3−^ (for 1 ppm and 10 ppm concentrations). As shown in Figure 9a, the removal efficiency was slightly hindered by the presence of anionic species; however, except for the phosphate ions, Chi@Fe_2_O_3_–PVDF still had approximately 80% removal efficiency in 1 ppm of coexisting ion. This is because all anions have electrostatic interactions with the amino groups in protonated chitosan. However, although Cl^−^, NO_3_^−^, and SO_4_^2−^ ions exhibit weaker adsorption mechanisms than Cr(VI), PO_4_^3−^ is an anchoring group and has an affinity with iron oxide; thus, its effect on the removal efficiency is more dramatic [39,40]. In addition, as the concentration of coexisting ions increased to 10 ppm, the competitive effect of the coexisting ions on Cr(VI) slightly increased. Further, the desalinization process was applied to real water samples such as tap water, drinking water, and river water. As shown in Figure 9b, the removal efficiency was slightly decreased compared to that of the control (solution in deionized water), but there was no noticeable difference. There are several ions in the actual water samples that compete with Cr(VI); however, in general, there was no significant change in adsorption efficiency for the various types of water samples.

There have been several reports about Cr(VI) removal methods using functionalized nanomaterials. In general, these materials have been applied in the batch system for removal of target metal ions and thus, the metal-containing adsorbents should be recovered from water after the purification procedure was accomplished. The nanoadsorbents-embedded composite membrane used in this study does not require the recovery of adsorbents from water, and therefore, this approach can provide a more convenient purification procedure than the batch process. It should be noted that with a single filtration process using Chi@Fe_2_O_3_–PVDF, Cr(VI) can be quickly removed with high efficiency, rendering this system as a method of choice for efficient water treatment (Table S1).

## 4. Conclusions

In this study, a new adsorption membrane was fabricated by immobilizing chitosan-coated iron oxide on a hydrophilic poly(vinylidene fluoride) membrane to effectively remove toxic substances in an aqueous solution. Through SEM-EDX analysis, it was confirmed that nanoparticles were successfully immobilized in the PVDF membrane sheet. The optimized pH condition with the highest removal efficiency for the Cr(VI) of Chi@Fe_2_O_3_–PVDF was pH 4, and pretreated Chi@Fe_2_O_3_–PVDF showed a higher removal efficiency than that of general Chi@Fe_2_O_3_–PVDF under acidic conditions. The Chi@Fe_2_O_3_–PVDF exhibited 14.104 mg/g of adsorption capacity in continuous in-flow conditions. In the presence of competing ions, there was a slight decrease in the Cr(VI) removal efficiency of Chi@Fe_2_O_3_–PVDF, except for PO_4_^3−^ ions that interact with both the chitosan functional groups and the iron oxide nanoparticles. The removal efficiency in the real water samples was high without any significant differences from the Cr(VI) removal efficiency in deionized water. These results indicate that Chi@Fe_2_O_3_–PVDF has high potential for removal of heavy metals from aqueous solutions.

## Figures and Tables

**Table 1:** Quantification of detached iron oxide nanoparticles under acidic, basic, and high-salt conditions after 1 h, analyzed by inductively coupled plasma spectrometer.

Solvent	Chi@Fe_2_O_3_–PVDF	
	ppm (mg/L)	Wt%
Deionized water	N.D.	-
Cr(VI) 25 ppm (pH 4)	N.D.	-
Cr(VI) 25 ppm (pH 7)	0.131	0.033%
Cr(VI) 25 ppm (pH 10)	0.182	0.046%
0.1 M HCl	1.469	0.371%
0.1 M NaOH	N.D.	-
0.1 M NaCl	N.D.	-

**Table 2:** Adsorption isotherms and kinetic parameters of Cr(VI) onto Chi@Fe_2_O_3_–PVDF.

System	Langmuir Constants		Freundlich Constants				
	K_L_ (L mg^−1^)	Q_max_ (mg g^−1^)	R^2^	K_F_ (mg g^−1^) (L mg^−1^)^1/n^		n	R^2^
Batch	1.640	14.451	0.998	7.036		3.670	0.571
Continuous-Flow	18.658	14.104	0.995	11.454		6.337	0.527
**System**	**Experimental value**	**Pseudo-first order**			**Pseudo-second order**		
	Q_e,exp_ (mg g^−1^)	k_1_ (min^−1^)	Q_e,cal_ (mg g^−1^)	R^2^	k_2_ (g mg^−1^ min^−1^)	Q_e,cal_ (mg g^−1^)	R^2^
Batch	28.737	0.011	9.206	0.3748	0.634	21.231	0.9994

## Data Availability

The data presented in this article are available on request from the corresponding authors.

## Supplementary Materials

The following supporting information can be downloaded at https://www.mdpi.com/article/10.3390/toxics10020098/s1, Figure S1: Procedure for the preparation of the composite membrane (Chi@Fe_2_O_3_-PVDF) via a vacuum filtration; Figure S2: Linear relationship of the absorbance at 540 nm with the concentrations of Cr(VI) in water by the standard diphenylcarbazide method; Figure S3: Linear relationship of the absorbance at 370 nm with the concentrations of Cr(VI) in water by direct method; Figure S4: Adsorption isotherm for continuous in-flow experiment (a) Langmuir model, (b) Freundlich model; Table S1: Comparison of other adsorption methods for the removal of Cr(VI) from aqueous solution.
