# Supplementary material for: Removal of Hexavalent Chromium(VI) from Wastewater Using Chitosan-Coated Iron Oxide Nanocomposite Membranes
Source: Toxics. 2022 Feb 19;10(2):98. doi: 10.3390/toxics10020098 (PMC8875893; doi:10.3390/toxics10020098)
Supplement: Supplementary file 1 [file toxics-10-00098-s001.zip › toxics-1556592-supplementary.pdf]

toxics

Supplementary Materials: Removal of Hexavalent Chromium(VI) from Wastewater Using Chitosan-coated Iron Oxide Nanocomposite Membranes

Jung Eun Park, Jun-Ho Shin, Wonzin Oh, Sang-June Choi, Jeongju Kim, Chorong Kim and Jongho Jeon

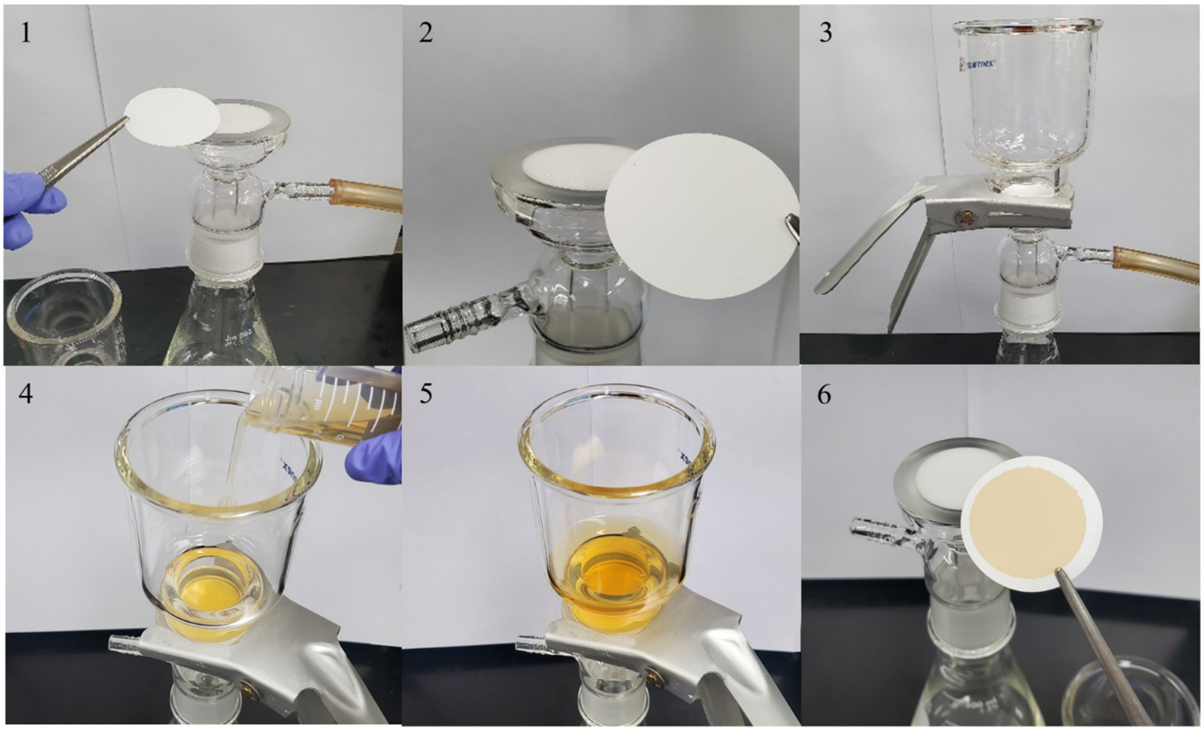

Figure S1. Procedure for the preparation of the composite membrane (Chi@Fe_2O_3-PVDF) via a vacuum filtration.

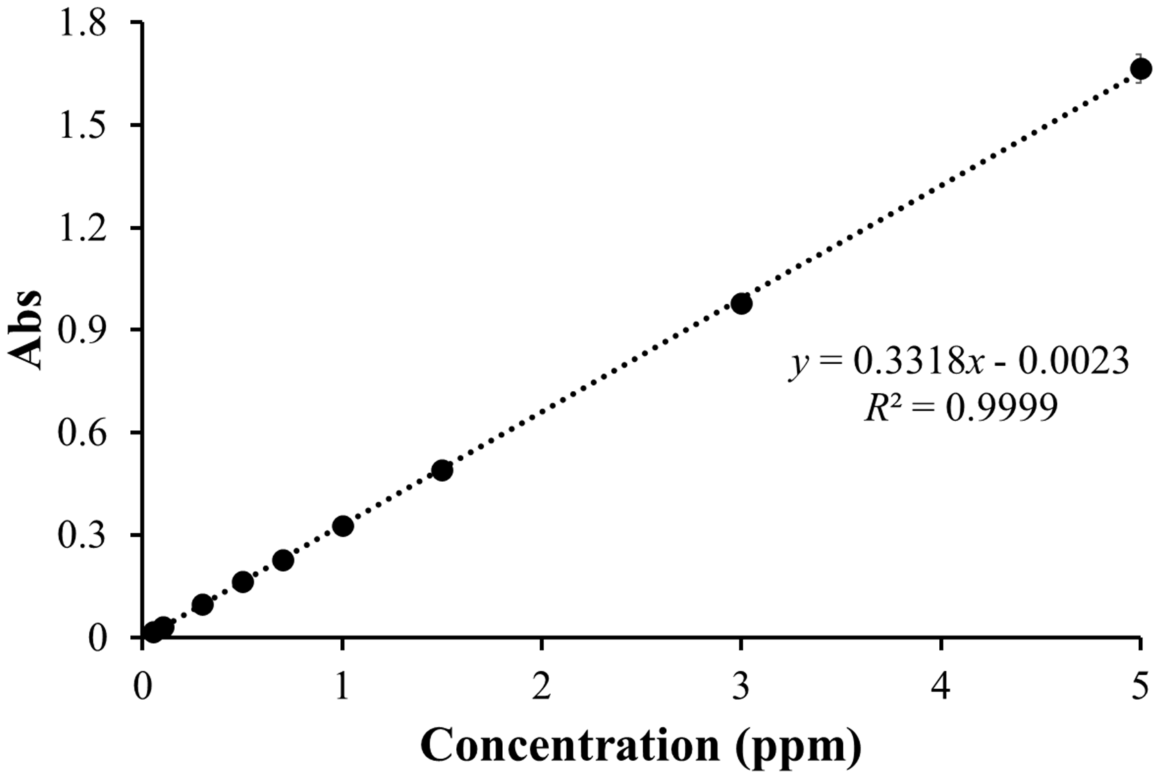

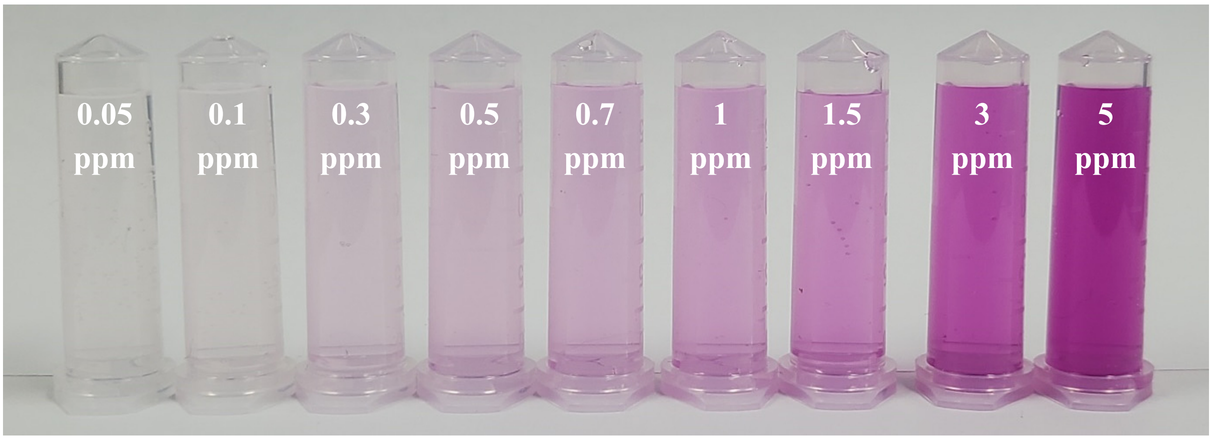

Figure S2. Linear relationship of the absorbance at 540 nm with the concentrations of Cr (VI) in water by the standard diphenylcarbazide method.

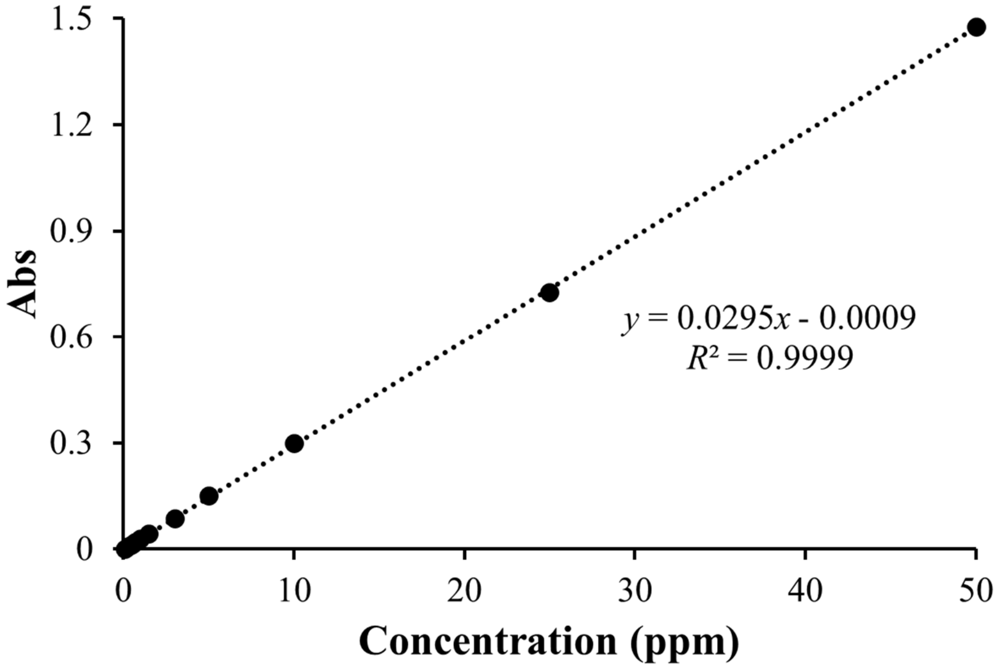

Figure S3. Linear relationship of the absorbance at 370 nm with the concentrations of Cr (VI) in water by direct method.

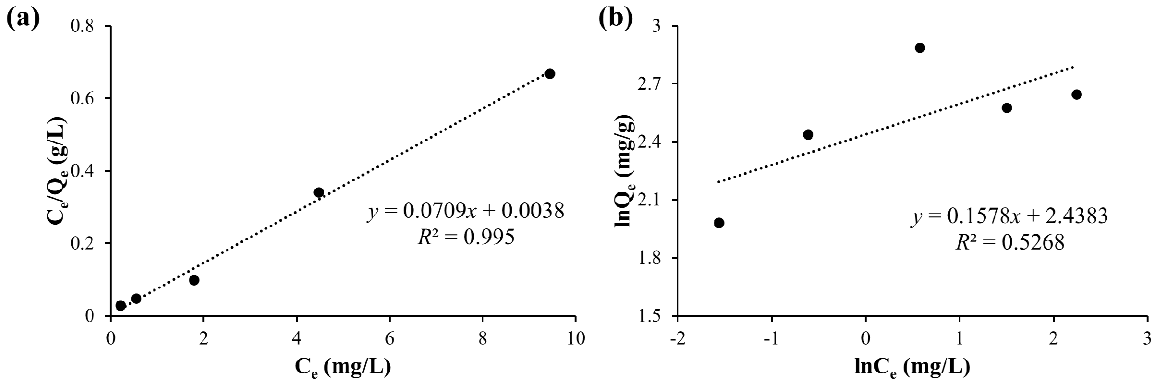

Figure S4. Adsorption isotherm for continuous in-flow experiment (a) Langmuir model, (b) Freundlich model.

Table S1. Comparison of other adsorption methods for the removal of Cr(VI) from aqueous solution.

Adsorbent	Contact time (min)	Adsorption capacity (mg/g)	Ref
GO/MNO_2/Fe_3O_4/Polypyrrole	1500	374.53	1
Protonated crosslinked chitosan	60	189.3	2
Deacetylated chitosan-coated magnetic adsorbent	80	24.66	3
Chi@Fe_3O_4	250	142.38	4
Chi@Fe_3O_4GO	250	100.51	4
Polypyrrole/OMWCNTs NCs	300	294.18	5
chitosan cross-linked with *N,N*−methylene-bis-acrylamide	60	149	6
**Chi@Fe_2O_3-PVDF**	**0.25**	**14.1**	**This work**

References

1. Liu, W.; Yang, L.; Xu, S.; Chen, Y.; Liu, B.; Li, Z.; Jiang, C. Efficient removal of hexavalent chromium from water by an adsorption-reduction mechanism with sandwiched nanocomposites. *RSC Adv.* **2018**, *8*, 15087–15093, doi: 10.1039/C8RA01805G
2. Huang, R.; Yang, B.; Liu, Q. Removal of chromium(VI) Ions from aqueous solutions with protonated crosslinked chitosan. *J. Appl. Polym. Sci.* **2013**, *129*, 908–915, doi: 10.1002/app.38685
3. Ravi, T.; Anuradha Jabasingh, S. Preparation and characterization of higher degree-deacetylated chitosan-coated magnetic adsorbent for the removal of chromium(VI) from its aqueous mixture. *J. Appl. Polym. Sci.* **2017**, *135*, 45878, doi: 10.1002/app.45878
4. Subedi, N.; Lähde, A.; Abu-Danso, E.; Iqbal, J.; Bhatnagar, A. A comparative study of magnetic chitosan (Chi@Fe3O4) and graphene oxide modified magnetic chitosan (Chi@Fe3O4GO) nanocomposites for efficient removal of Cr(VI) from water. *Int. J. Biol. Macromol.* **2019**, *137*, 948–959, doi: 10.1016/j.ijbiomac.2019.06.151
5. Bhaumik, M.; Agarwal, S.; Gupta, V.K.; Maity, A. Enhanced removal of Cr(VI) from aqueous solutions using polypyrrole wrapped oxidized MWCNTs nanocomposites adsorbent. *J. Colloid Interface Sci*. **2016**, *470*, 257–267, doi: 10.1016/j.jcis.2016.02.054
6. Ismael, M.N.M.; El Nemr, A.; El Ashry, E.S.H.; Hamida, H.A. Removal of Hexavalent Chromium by Cross-Linking Chitosan and *N,N'*-Methylene Bis-Acrylamide. *Environ. Process.* **2020**, *7*, 911–930, doi: 10.1007/s40710-020-00447-2
